# Comparison of health outcomes between traumatic spinal cord and cauda equina injuries

**DOI:** 10.1038/s41393-026-01191-4

**Published:** 2026-03-13

**Authors:** Xenia Beaumont, Susan Liew, Sandra Reeder, Joanna F. Dipnall, Belinda Gabbe

**Affiliations:** 1 School of Public Health and Preventive Medicine, Monash University, Melbourne, VIC Australia; 2 Monash Department of Surgery, The Alfred, Melbourne, VIC Australia; 3 Department of Orthopaedic Surgery, The Alfred, Melbourne, VIC Australia; 4 Monash Centre for Health Research and Implementation, Monash University, Melbourne, VIC Australia; 5 School of Medicine, Deakin University, Melbourne, VIC Australia

**Keywords:** Epidemiology, Trauma

## Abstract

**Study design:**

Retrospective cohort study of prospectively registered and collected data.

**Objectives:**

To compare the outcomes of people with a traumatic spinal cord injury (SCI) or cauda equina injury (CEI).

**Setting:**

Victorian State Trauma System, Australia.

**Methods:**

People from the Victorian State Trauma Registry (VSTR) with a diagnosis of traumatic SCI below T4 or CEI, with a date of injury from 2010 to December 2022, were included. Participants were divided into two groups; upper motor neuron (UMN) and lower motor neuron (LMN) injury groups. Demographic, injury event, and hospital details were extracted. Follow-up was conducted by the registry up to 24-months post injury, including the World Health Organization Disability Assessment Schedule (WHODAS), EuroQol EQ-5D scale, and return to work.

**Results:**

Of the 1156 participants, 1113 were categorised as UMN, and 43 as LMN. Within both groups, the demographics were similar. Mixed effect regression modelling of the EQ-5D outcomes over time showed little improvement for both groups. The return to work rate was higher for the LMN injury group at all follow-up time points, with 57% of the UMN injury group returning to work at 24 months post-injury compared to 72% of the LMN injury group. At 24 months post-injury, 63% of people in the UMN injury group reported a WHODAS score equal or greater to ten, compared to 65% in the LMN injury group.

**Conclusion:**

The patient demographics within the two groups were similar. Overall quality of life outcomes remained similarly poor over time for both groups, with little improvement.

## Introduction

The incidence of Spinal Cord Injury (SCI) in Australia is estimated to be between 21 and 32.3 per million per year and costs Australia $3.7 billion annually, reflecting the significance and severity of these injuries [1, 2]. A SCI or cauda equina injury (CEI) can occur following trauma, such as a road traffic crash or a fall [3]. Injuries to the spinal cord or conus medullaris are upper motor neuron (UMN) injuries and form part of the central nervous system. The cauda equina is the continuation of the end of the spinal cord, and forms part of the lower motor neuron (LMN) or peripheral nervous system. The presentation of both UMN and LMN injuries differs, as does the potential degree of recovery [4, 5].

The overall neurological impacts of these injuries can be devastating, and can result in impairment of the motor, sensory, and autonomic systems, depending on the level and extent of injury [6, 7]. Motor dysfunction can result in weakness or paralysis, sensory dysfunction can result in paraesthesia and anaesthesia, and autonomic dysfunction encompasses issues with bowel and bladder sphincter control, sexual function, autonomic dysreflexia, cardiovascular and respiratory function [6, 7]. These physical impairments can then impact on overall quality of life, mental health and participation in society [8].

Whilst SCI outcomes due to trauma have been documented, few studies have compared outcomes between higher and lower levels of traumatic SCI [3, 9–12]. Several studies reported a greater degree of neurological recovery in people with CEI and/or conus medullaris injury (CMI), than people with a higher-level SCI [3, 10, 12]. However, these findings differ to a cohort study by Kaneda et al., which reported greater improvement in motor outcomes in people with a SCI than people with a CMI or CEI [11]. Some studies have also shown no difference in overall functional or health quality of life outcomes between the different levels of SCI and CEI [3, 12]. The aim of this population-based study was to elucidate the epidemiology and longer-term outcomes of people with a traumatic UMN and LMN spinal injury. The hypothesis is that people with a traumatic LMN spinal injury should have a greater level of recovery than people with an UMN injury.

## Methods

### Setting

This was a retrospective cohort study of prospectively registered and collected data. Data from the population-based, Victorian State Trauma Registry (VSTR) was used. The VSTR was established in 2001 and collects data on all major trauma patients from the state of Victoria in Australia, which has a population of 6.9 million people [13, 14]. The VSTR collects data from all 138 trauma receiving hospitals in the Victorian State Trauma System (VSTS), including the three designated major trauma services (Level 1 trauma centre equivalent) [15]. Provision of data to the VSTR is mandatory for trauma-receiving hospitals. The major trauma inclusion criteria include death following injury, an Injury Severity Score (ISS) score greater than 12, 24 h or greater length of intensive care unit (ICU) stay and requiring mechanical ventilation, urgent surgery, and burns equal or greater to 20% of the total body surface area [16]. In the VSTS, Ambulance Victoria provides ambulance transport and retrieval of trauma patients, with patients triaged according to trauma guidelines [17]. The VSTR has ethics approval from the Department of Health and Department of Families, Fairness and Housing Human Research Ethics Committee to operate this registry. Ethics approval for this study was given by the Monash University Human Research Ethics Committee (project number 42181, approval date: 09/04/2024). This study only used non-identifiable data from a registry, thus informed consent was not sought from participants.

### Participants and procedures

All major trauma patients in Victoria are assigned both an Abbreviated Injury Scale (AIS) 2008 revision, and an International Statistical Classification of Diseases and Related Health Problems, Tenth Revision, Australian Modification (ICD-10-AM) code. The AIS is collected by train health coders at individual hospital sites or by Monash University. The ICD-10-AM is routine coding undertaken for all hospital admissions and is done by trained coders employed by individual health services.

### Participants were included in this study if they met the following criteria

I. Adults aged 16 years and over.
II. Injury date from January 2010 to December 2022.
III. An AIS or ICD-10-AM code for any spinal cord injury at or below T4 and patients with a cauda equina injury diagnosis, were included in this study (see supplementary materials Appendix 1 for specific injury codes).

### Participants were excluded if the following were present

I. A concomitant injury to the cervical spinal cord based on AIS and ICD-10-AM codes and text descriptors.
II. A code for lumbar spinal cord injury without a level of injury specified.

Patients with a SCI from L1 to T4 inclusive, or conus medullaris injury, were assigned to the upper motor neuron (UMN) injury group. Patients with a code for cauda equina or participants coded as having a spinal cord injury L2 and below were assigned to the lower motor neuron (LMN) injury group. A cut-off of L2 has been used by similar studies, and the L1 region has been described as the median location for the conus medullaris in adults [9, 18]. People who had concomitant UMN injury and an LMN injury were assigned to the UMN injury group, as clinically these patients would exhibit UMN signs [19].

The data extracted from the VSTR included demographic, injury event, hospital stay and patient-reported outcome variables. Participants were classified to metropolitan or regional area of residence based on their residential postcode and the Accessibility/Remoteness Index of Australia (ARIA). [20] Participants’ residential postcodes were also assigned an Index of Relative Socio-Economic Advantage and Disadvantage (IRSAD) quintile, reflecting the level of disadvantage or advantage of certain areas [21]. The Charlson Comorbidity Index (CCI) reflects a person’s mortality risk due to a set of specific comorbidities based on ICD-10-AM diagnosis codes [22]. A CCI of zero reflects a person with no CCI conditions, whilst a higher CCI weight reflects an increasing risk of death. The CCI weight was categorized to zero, one, or higher than one. The ISS reflects a person’s overall injury severity, with scores ranging from one to 75 [23]. The registry also collects information about procedures and surgeries that are performed for each person. The registry receives the Australian Classification of Health Interventions (ACHI) procedure code and registry coders also allocate Victorian State Trauma Outcomes Registry and Monitoring Group (VSTORM) procedure codes, which reflect the treatment they received in hospital for their injuries. The Australian Classification of Health Interventions and VSTORM procedure codes were used to identify a relevant spinal operation, including spinal decompression, open and closed reductions, spinal fusions and internal fixation of the spine. This spinal surgery variable was collapsed into a binary variable, with patients classified as either receiving or not receiving spinal surgery during their hospital stay. The length of stay variable categories were based on quartiles.

Routine follow-up occurs at six, 12- and 24-months post injury by registry staff, using either telephone or online questionnaires [24]. A proxy may be used if the patient is unable to complete the interview themselves. Several phone calls are attempted before a person is deemed to be lost to follow-up. Deaths following hospital discharge are identified by linkage with the Victorian Registry of Deaths, Births and Marriages.

A commonly used and validated tool to explore health status is the EuroQol EQ-5D, which collects information about mobility, self-care, usual activities, pain and/or discomfort, and anxiety and/or depression [25]. The VSTR collects EQ-5D outcomes at each follow-up time point. A three-level and a five-level version of the EQ-5D tool exist, with the VSTR previously using the three-level prior to July 2018. Thus, the five-level version was subsequently mapped to the three-level version to allow for direct collation of data. The three-level version was further collapsed into two groups, due to low cell counts. The VSTR also administers the 12-item WHODAS 2.0 survey, which reflects a patient’s overall level of disability, reflecting the level of disability in the following categories: cognition, mobility, self-care, social interaction, general life activities and social participation [26]. Participants are asked to answer question pertaining to these categories following their experience in the previous 30 days. An overall WHODAS score is assigned to each participant, between zero and 48, with higher scores representing greater disability. In this study the total WHODAS score variable was collapsed into “<10” and “10 + ”, with the “10 + ” score signifying a greater level of disability, and “<10” representing a less severe level of disability [27]. Return to work outcomes are also captured in the follow-up, with patients asked to respond either “yes” or “no” to whether they had returned to work/study, whether they returned to the same workplace, and whether they returned to the same role at the same organization.

### Data analysis

Descriptive statistics were used to summarise data. Categorical variables were summarised using frequencies and percentages, whilst normally distributed continuous variables were presented with the mean and standard deviation (SD), and the median and interquartile range (IQR) for non-normal distribution, with missing data highlighted in the tables for each variable. For each point of follow-up of the EQ-5D outcomes, predicted probabilities and 95% confidence intervals were calculated using mixed effects logistic regression modelling with random intercepts. The variables included in the mixed effects logistic regression modelling included the five EQ-5D domain categories and follow-up time, as well as spinal surgery, a binary variable demonstrating whether a participant had spinal surgery during their hospital stay, as a fixed effect. This variable was chosen as it reflects injury severity, such as instability and degree of neurological involvement, and is a known confounder for spine-related outcomes. The output of these models is presented in Appendix 2. Chi square tests were used to compare demographic variables, injury characteristics and outcome variables between the two injury groups, with their corresponding bootstrapped p-values and BCa 95% confidence intervals reported. As there was a substantial difference in the number of UMN and LMN cases, the disproportion between groups limited the power of the study, and bootstrapping was applied to the Chi square test of independence, with 2000 replications and a random seed set [28]. A p-value of <0.05 was considered statistically significant. For statistical analysis, Stata Version 17 (StataCorp, College Station, TX) was used, with all analysis performed in the Monash Secure eResearch Platform (SeRP).

## Results

### Cohort overview

**Fig. 1:**
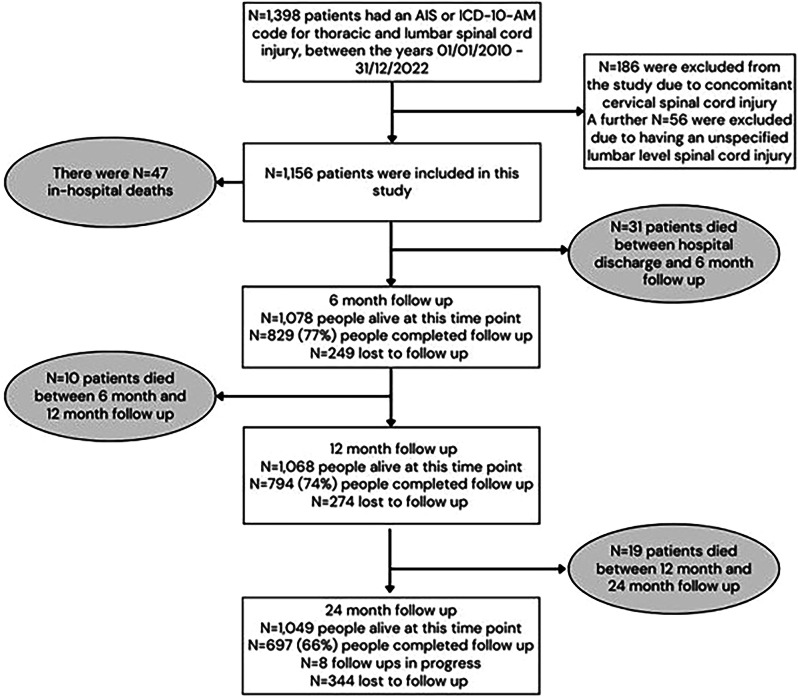
This figure shows the number of participants eligible for the study, as well as follow-up completion rates at each follow-up time point.

There were 1156 participants on the VSTR who met the inclusion criteria for this study. Of these people, 47 died during their hospital stay (Fig. 1). The median age of people in the UMN group was 47 years of age, and was 50 years of age for the LMN group. Table 1 shows the key demographic characteristics of the cohort. The cause of injury, whether participants had spinal surgery during their initial hospitalisation, and their discharge destination were found to be statistically significant.

**Table 1:** Demographics table of N = 1156 patients.

Variable		UMN injury *N* = 1113 (%)	LMN injury *N* = 43 (%)	Total cohort *N* = 1156 (%)	*P*-value	BCa 95% Confidence Interval
Age category	16–39 years	484 (44%)	19 (44%)	503 (44%)	0.93	(0.00, 0.31)
	40–59 years	287 (26%)	10 (23%)	297 (26%)		
	60+ years	342 (31%)	14 (33%)	356 (31%)		
Sex	Male	778 (70%)	30 (70%)	808 (70%)	0.99	(0.00, 0.00)
	Female	335 (30%)	13 (30%)	348 (30%)		
Level of education attained^a^	At high school or not completed	302 (34%)	9 (26%)	311 (34%)	0.95	(0.26, 2.65)
	Advanced diploma/certificate	228 (26%)	11 (32%)	239 (26%)		
	Completed high school	150 (17%)	7 (21%)	157 (17%)		
	University	171 (19%)	6 (18%)	177 (19%)		
	Unknown or other	34 (4%)	1 (3%)	33 (4%)		
Metropolitan or regional area of residence	Metropolitan area	647 (58%)	30 (70%)	677 (59%)	0.45	(0.37, 8.20)
	Regional area	364 (33%)	10 (23%)	374 (32%)		
	Interstate/overseas/no fixed abode/unknown	102 (9%)	3 (7%)	105 (9%)		
Socioeconomic Status (IRSAD)^b^	1 (most disadvantaged)	154 (14%)	5 (12%)	159 (14%)	0.93	(0.15, 1.16)
	2	199 (18%)	7 (16%)	206 (18%)		
	3	222 (20%)	9 (21%)	231 (20%)		
	4	244 (22%)	12 (28%)	256 (23%)		
	5 (least disadvantaged)	265 (24%)	10 (23%)	275 (24%)		
Charlson Comorbidity Index weight (CCI)	0	727 (65%)	25 (58%)	752 (65%)	0.60	(0.00, 5.46)
	1	248 (22%)	11 (26%)	259 (22%)		
	>1	138 (12%)	7 (16%)	145 (13%)		
Cause of injury	Road trauma	485 (44%)	10 (23%)	495 (43%)	0.006	(2.28, 26.26)
	High falls	273 (25%)	15 (35%)	288 (25%)		
	Low falls	189 (17%)	14 (33%)	203 (18%)		
	Other	166 (15%)	4 (9%)	170 (15%)		
Injury Severity Score > 12^c^	No	261 (26%)	16 (38%)	277 (26%)	0.07	(0.02, 16.84)
	Yes	761 (74%)	26 (62%)	787 (74%)		
Spinal surgery during hospital stay	No	634 (57%)	14 (33%)	648 (56%)	0.002	(1.24, 23.34)
	Yes	479 (43%)	29 (67%)	508 (44%)		
Length of hospital stay in quartiles	Q1 0 to 5.9 days	293 (26%)	11 (26%)	304 (26%)	0.38	(0.07, 8.50)
	Q2 6 to 9.9 days	245 (22%)	9 (21%)	254 (22%)		
	Q3 10 to 18.9 days	305 (27%)	8 (19%)	313 (27%)		
	Q4 19 to 292 days	270 (24%)	15 (35%)	285 (25%)		
Intensive care unit stay^d^	No ICU stay	815 (73%)	36 (84%)	851 (74%)	0.13	(0.00, 8.27)
	ICU stay	297 (27%)	7 (16%)	304 (26%)		
Discharge destination	Rehabilitation	563 (51%)	20 (47%)	583 (51%)	0.03	(3.12, 39.06)
	Home	423 (38%)	15 (35%)	438 (38%)		
	Nursing home	10 (1%)	0 (0%)	10 (1%)		
	Hospital for Convalescence/other	70 (6%)	8 (18%)	78 (7%)		
	In hospital death	47 (4%)	0 (0%)	47 (4%)		

Missing data: a (n = 237); b (n = 29); c (n = 92); d (n = 1).

*CCI* Charlson Comorbidity Index, *Q* Quartile, *ICU* – intensive care unit, *IRSAD* Index of Relative Socio-economic Advantage and Disadvantage, *LMN* lower motor neuron, *UMN* upper motor neuron, *BCa* Bias-Corrected and Accelerated.

### Health status outcomes

**Fig. 2:**
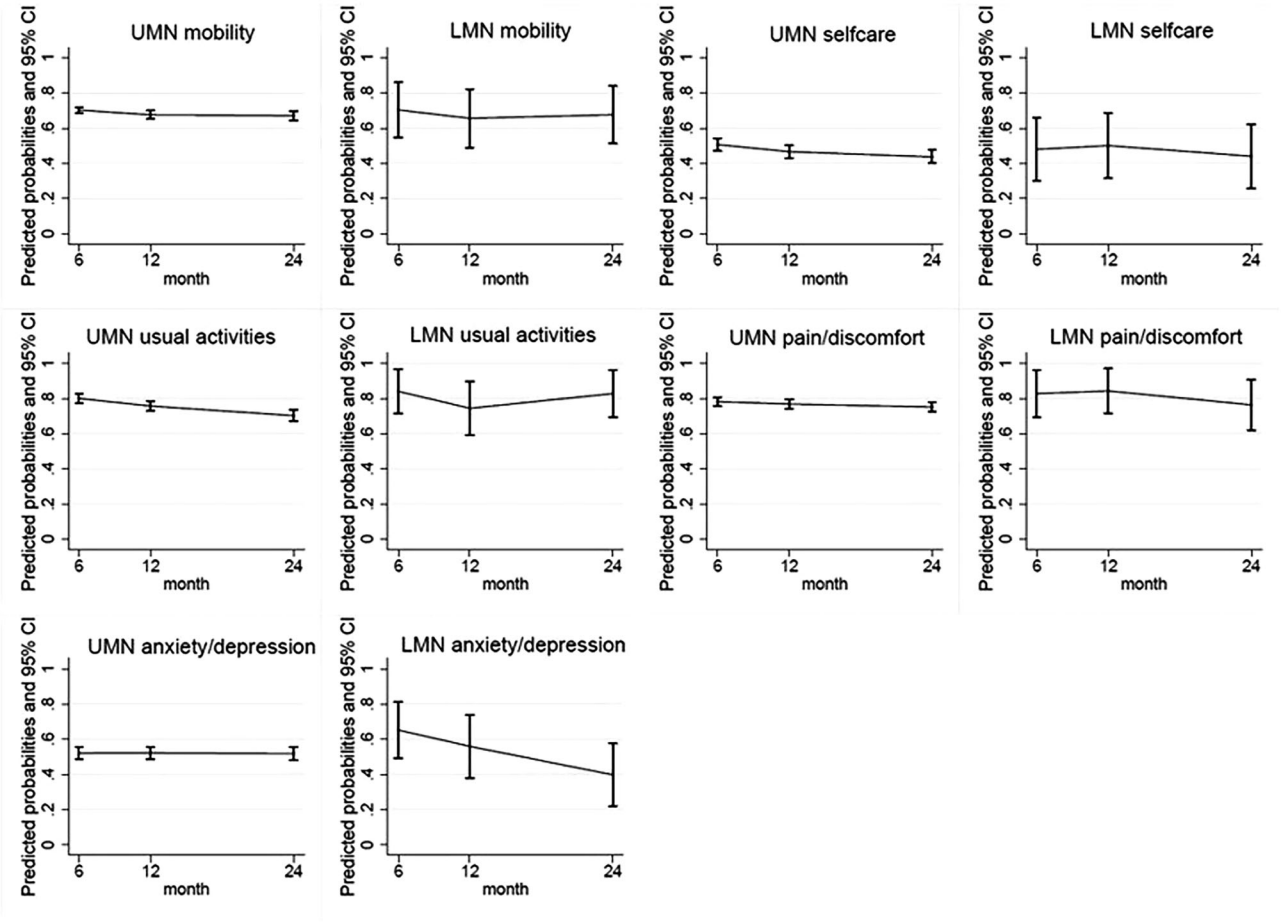
This figure shows the results from the mixed effects regression modelling of each EQ-5D item across 6-, 12-, and 24- months. Each graph displays the proportions and 95% confidence intervals at each time point. A graph is shown for each injury group for each EQ-5D item.

Of the survivors in the overall cohorts, N = 829 (77%) completed six-month follow-up, N = 794 (74%) completed 12-month follow-up and *N* = 697 (66%) completed 24-month follow-up (Fig. 1). Within the overall cohort at 24- months following injury, most reported some degree of problems on all the EQ-5D items except self-care (Table 2). The proportions of people reporting some degree of problems was similar across all EQ-5D items and time points with both the UMN and LMN injury groups. The proportion of participants reporting problems on the EQ-5D items did not change over time, overall or between groups (Fig. 2). The percentage of people scoring 10 or more on the WHODAS, signifying a greater level of disability, was marginally higher in the LMN injury group than the UMN injury group at all follow-up time pointsbut this difference was not statistically significant (Table 3). Six-hundred forty-two people were working or studying prior to their injury; return to work rates were higher in the LMN injury group than the UMN injury group across all follow-up times points (65 and 45% at six- months, 67 and 54% at 12- months, and 72 and 57% at 24- months for the LMN and UMN injury groups respectively) (Table 4). Of the participants that did return to work, most people in the LMN injury group returned to the same organisation. Within both injury groups, most people returned to the same role if they did return to work.

**Table 2:** EQ-5D outcomes at follow-up.

	UMN injury	LMN injury neuron injury	Overall	P value	BCa 95% Confidence Interval*	UMN injury	LMN injury neuron injury	Overall	P value	BCa 95% Confidence Interval*	UMN injury	LMN injury neuron injury	Overall	P value	BCa 95% Confidence Interval*
Total respondents (N)	N = 797	N = 32	N = 829			N = 764	N = 30	N = 794			N = 667	N = 30	N = 697		
	Mobility 6 months^a^					Mobility 12 months^b^					Mobility 24 months^c^				
No problems	261 (33%)	9 (28%)	270 (33%)	0.57	(0, 3.07)	289 (38%)	11 (37%)	300 (38%)	0.89	(0.00, 0.15)	259 (39%)	10 (33%)	269 (39%)	0.54	(0.00, 3.57)
Some degree of problems	532 (67%)	23 (72%)	555 (67%)			474 (62%)	19 (63%)	493 (62%)			407 (61%)	20 (67%)	427 (61%)		
	Self-care 6 months^d^					Self-care 12 months^e^					Self-care 24 months				
No problems	397 (50%)	16 (50%)	413 (50%)	0.99	(0,0)	413 (54%)	16 (53%)	429 (54%)	0.93	(0, 0.04)	378 (57%)	17 (57%)	395 (57%)	0.99	(0, 0)
Some degree of problems	399 (50%)	16 (50%)	415 (50%)			350 (46%)	14 (47%)	364 (46%)			289 (43%)	13 (43%)	302 (43%)		
	Usual activities 6 months^f^					Usual activities 12 months					Usual activities 24 months^g^				
No problems	159 (20%)	5 (16%)	164 (20%)	0.54	(0, 3.13)	186 (24%)	8 (27%)	194 (24%)	0.77	(0, 1.06)	198 (30%)	5 (17%)	203 (29%)	0.12	(0.01, 8.07)
Some degree of problems	635 (80%)	27 (84%)	662 (80%)			578 (76%)	22 (73%)	600 (76%)			468 (70%)	25 (83%)	493 (71%)		
	Pain and/or discomfort 6 months^h^					Pain and/or discomfort 12 months^i^					Pain and/or discomfort 24 months^j^				
No problems	167 (21%)	5 (16%)	172 (21%)	0.45	(0.00, 3.96)	175 (23%)	5 (17%)	180 (23%)	0.42	(0.00, 4.43)	160 (24%)	7 (23%)	167 (24%)	0.92	(0, 0.05)
Some degree of problems	622 (79%)	27 (84%)	649 (79%)			586 (77%)	25 (83%)	611 (77%)			503 (76%)	23 (77%)	526 (76%)		
	Anxiety and/or depression 6 months^k^					Anxiety and/or depression 12 months^l^					Anxiety and/or depression 24 months^m^				
No problems	380 (48%)	11 (34%)	391 (48%)	0.12	(0.01, 11.26)	373 (49%)	14 (47%)	387 (49%)	0.79	(0, 0.73)	330 (50%)	18 (62%)	348 (50%)	0.19	(0.01, 9.75)
Some degree of problems	404 (52%)	21 (66%)	425 (52%)			386 (51%)	16 (53%)	402 (51%)			336 (50%)	11 (38%)	347 (50%)		

Missing values: a (n = 4); b (n = 1); c (n = 1); d (n = 1); e (n = 1); f (n = 3); g (n = 1); h (n = 8); i (n = 3); j (n = 4); k (n = 13); l (n = 5); m (n = 2).

*LMN* lower motor neuron, *UMN* upper motor neuron.

*BCa Bias-Corrected and Accelerated.

**Table 3:** WHODAS disability score.

6 months^a^					
	UMN injury N = 444	LMN injury N = 23	Overall N = 467	P value	BCa 95% CI*
Less severe level of disability (WHODAS < 10)	151 (34%)	7 (30%)	158 (34%)	0.72	(0, 1.48)
Greater level of disability (WHODAS ≥ 10)	293 (66%)	16 (70%)	309 (66%)		
12 months^b^					
	UMN injury N = 452	LMN injury N = 20	Overall N = 472	P value	BCa 95% CI*
Less severe level of disability (WHODAS < 10)	173 (38%)	7 (35%)	180 (38%)	0.77	(0,0.94)
Greater level of disability (WHODAS ≥ 10)	279 (62%)	13 (65%)	292 (62%)		
24 months^c^					
	UMN injury N = 449	LMN injury N = 20	Overall N = 469	P value	BCa 95% CI*
Less severe level of disability (WHODAS < 10)	168 (37%)	7 (35%)	175 (37%)	0.83	(0, 0.43)
Greater level of disability (WHODAS ≥ 10)	281 (63%)	13 (65%)	294 (63%)		

Missing values: a (n = 362); b (n = 322); c (n = 228).

*LMN* lower motor neuron, *UMN* upper motor neuron, Bias-Corrected and Accelerated.

**Table 4:** Return to work outcomes.

Return to work after injury																
	6 months^a^					12 months^b^					24months^c^					
	UMN injury neuron N = 545	LMN injury N = 20	Overall N = 565	P value	BCa 95% CI*	UMN injury N = 527	LMN injury N = 18	Overall N = 545	P value	BCa 95% CI*	UMN injury N = 487	LMN injury N = 18		Overall N = 505	P value	BCa 95% CI*
Returned to work	243 (45%)	13 (65%)	256 (45%)	0.07	(0.01, 13.18)	286 (54%)	12 (67%)	298 (55%)	0.30	(0, 7.11)	279 (57%)	13 (72%)		292 (58%)	0.21	(0, 7.64)
Did not return to work	302 (55%)	7 (35%)	309 (55%)			241 (46%)	6 (33%)	247 (45%)			208 (43%)	5 (28%)		213 (42%)		
Missing values: a (n = 77); b (n = 97); c (n = 137)																
Return to same organisation after injury																
	6 months^d^					12 months^e^					24months^f^					
	UMN injury N = 243	LMN injury N = 13	Overall N = 256	P value	BCa 95% CI*	UMN injury N = 284	LMN injury N = 12	Overall N = 296	P value	BCa 95% CI*	UMN injury N = 278	LMN injury N = 13		Overall N = 291	P value	BCa 95% CI*
Returned to same organisation	206 (85%)	13 (100%)	219 (86%)	0.13	(1.75, 3.41)	218 (77%)	12 (100%)	230 (78%)	0.06	(2.85, 4.80)	197 (71%)	11 (85%)		208 (72%)	0.28	(0, 5.25)
Did not return to same organisation	37 (15%)	0 (0%)	37 (14%)			66 (23%)	0 (0%)	66 (22%)			81 (29%)	2 (15%)		83 (29%)		
Missing values: d (n = 386); e (n = 346); f (n = 351)																
Return to same role after injury																
	6 months^g^					12 months^h^					24months^i^					
	UMN injury neuron N = 240	LMN injury N = 13	Overall N = 253	P value	BCa 95% CI*	UMN injury N = 285	LMN injury N = 12	Overall N = 297	P value	BCa 95% CI*	UMN injury N = 279	LMN injury N = 13	Overall N = 292		P value	BCa 95% CI*
Returned to same role	184 (77%)	10 (77%)	194 (77%)	0.98	(0, 0)	202 (71%)	10 (83%)	212 (71%)	0.35	(0, 4.61)	181 (65%)	9 (69%)	190 (65%)		0.75	(0, 0.97)
Did not return to same role	56 (23%)	3 (23%)	59 (23%)			83 (29%)	2 (17%)	85 (29%)			98 (35%)	4 (31%)	102 (35%)			

Missing values: g (n = 389); h (n = 345); i (n = 350).

*LMN* lower motor neuron, *UMN* upper motor neuron.

*BCa Bias-Corrected and Accelerated.

## Discussion

### Key findings

In this population-based study, there were important differences in the causality of UMN and LMN injuries. Road trauma was the leading cause of injury for the UMN injury group, while a low fall or fall from standing height was the leading cause for the LMN injury group. In our study, the overall quality of life outcomes is represented by the EQ-5D and WHODAS outcome measures, and show similarly poor outcomes between the two groups. When considering the EQ-5D items, there seems to be little improvement over time in the UMN injury group, and improvement in the LMN injury groups is unclear. Overall, there were higher rates of return to work in the LMN injury group than the UMN injury group at every time point, with a majority of people in both injury groups returned to work at 24- months.

Whilst our study found most UMN injuries were caused by road trauma, a low fall or fall from standing height was the leading cause for LMN injuries. This contrasts with a retrospective cohort study of traumatic thoracolumbar spinal injuries by Kingwell et al, that found thoracic SCI below T11 inclusive and CMI were mostly due to falls (47 and 50% respectively), whilst CEI were caused equally by road traffic incidents (42%) and falls (42%) [3]. However, our results are consistent with the broader Victorian major trauma cohort, where the leading cause of major trauma is low falls in people aged over 64 years of age, followed by road trauma and falls from a height of more than one metre [16].

The EQ-5D tool has not been used in other comparative studies, however, differences in mobility have been reported on previously [3, 11, 12]. The current article showed little improvement in the EQ-5D item of mobility over time for both injury groups, with most reporting some degree of problems at all time points. This contrasts with the existing literature, where overall, people with CEI and/or CMI had better motor recovery than people with SCI. Kingwell et al. described worse motor improvement for people with a SCI and CEI than for people with a CMI, using a classification system assigning injury groupings based on MRI localisation of the Conus Medullaris. However, when these authors classified the level of injury based on vertebral levels, Kingwell et al. found no difference in motor improvement between these three groups [3]. Similarly, a prospective cohort study by Brouwers et al. described worse motor recovery for people with SCI than those with conus medullaris syndrome (CMS), and reported a difference in outcome of mobility, using the Spinal Cord Independence Measure (SCIM) sub-score for mobility indoors for SCI and cauda equina syndrome (CES), and between CMS and CES [12]. However, a retrospective cohort study by Kaneda et al. found better motor recovery in people with SCI than with CEI, however the motor score was worse at the final follow-up in people with SCI than people with CEI [11].

There is no clear consensus from the literature in regard to overall neurological outcomes between different levels of injury, and comparison is difficult due to the use of disparate tools. In our study, the WHODAS provides an overall disability measure, and shows little improvement over time for both injury groups. Similarly, the EQ-5D outcomes show little improvement for the UMN injury group, with outcomes unclear for the LMN injury group, however in both groups, a majority reported some degree of problems on all the EQ-5D items except self-care. A retrospective cohort study by Hashimoto et al. described worse overall neurological outcomes for people with epiconus lesions than people with cous medullaris or cauda equina lesions within two months of follow-up post-injury [10]. These findings contrast to those of Brouwers et al., who reported no difference in overall neurological outcomes using the SCIM between people with CES, CMS and SCI [12]. Similarly, a retrospective cohort study of different SCI syndromes by McKinely et al. found no difference in outcomes using the Functional Independence Measure score, rehabilitation length of stay or discharge disposition between patients with cauda equina syndrome and CMS, however these outcomes cannot be attributed specifically to a traumatic aetiology, as this study included patients with non-traumatic causes of injury [29]. A narrative literature review by Kingwell et al. also concluded that traumatic CEI and CMI have similar neurological outcomes [9]. Overall, previous articles in the literature report either no difference in overall neurological outcomes between different groups or in those that do report a difference in outcomes, it seems that SCI neurological outcomes are worse than outcomes reported for CMI and/or CEI.

The current article demonstrated most people returned to work at 24- months after injury in both groups, with higher rates of return in the LMN injury group. Comparison to existing literature is challenging, as to the authors’ knowledge, no comparison between traumatic SCI groups and return to work outcomes has been described.

### Strengths

Our study is a novel contribution to the literatures. Firstly, the unique grouping of comparing spinal cord lesions to lower motor neuron lesions of the cauda equina, presents a distinct method of comparing different levels of spinal injury. By separating people with CEI from true spinal cord lesions, we were better able to compare the recoverability of the peripheral nervous system compared to the central nervous system. It is hypothesised peripheral nervous system injuries have a better ability to recover than central nervous system injuries [30]. Other key strength are the use of a population-based cohort, the longitudinal design, and comparison across multiple patient-centred outcomes.

### Limitations

Our study had several limitations. Whilst attempts were made to include patients with a functional spinal injury below the level of T4, there may be a small number of patients who have had a concomitant higher-level thoracic injury. Comparison between the UMN and LMN injury groups was difficult due to an imbalance between the two groups, with a low prevalence of LMN injury in the overall cohort. A limitation of this study is the L1 vertebral level cut-off for assigning participants to either the upper or lower motor neuron injury groups. The L1 cut-off was based upon the reported average location of the conus medullaris [18, 31–34]. Furthermore, the L1 vertebral level was the most common level for the conus medullaris in five studies [31, 35–38]. However, its termination is variable, with a range of vertebral levels T11/12 to L3 reported in the literature [31–33, 35–37]. As a result, it is possible some cases of isolated cauda equina injuries were thus misclassified as upper motor neuron injuries in our study. The effect of the misclassification may dilute the differences between the two group, affecting result validity and potentially bias the results towards the null. The sample size for this study limited the number of potential confounding variables to be included in the mixed effects regression models. However, the inclusion of spinal surgery variable as a fixed effect, accounted for injury severity and the baseline difference between the two injury groups with regards to spinal surgery.

Further research is needed to more conclusively draw conclusions around recovery between different levels of spinal cord injury. In particular, consistent definitions and allocations of participants to SCI, CMI and CEI groupings are needed to enable better comparison. Most of the current research has focussed on motor recovery, with more research needed on other known complications including bowel, sex and sensory dysfunction.

## Conclusion

In conclusion, the demographics of patients within the UMN and LMN injury groups were similar except for the cause of injury. Overall health quality of life outcomes remained similarly poor over time for both injury groups, with little improvement in outcomes over time.

## Supplementary information

Supplementary material

## Data Availability

Data is available from the corresponding author upon reasonable request.

## Code availability

Stata Version 17 code is available upon reasonable request.
