# Supplementary material for: Comparison of health outcomes between traumatic spinal cord and cauda equina injuries
Source: Spinal Cord. 2026 Mar 13;64(4):337–45. doi: 10.1038/s41393-026-01191-4 (PMC13065472; doi:10.1038/s41393-026-01191-4)
Supplement: Supplementary file 1 — Supplementary material [file 41393_2026_1191_MOESM1_ESM.docx]

Supplementary material

Appendix 1: AIS and ICD-10-AM injury codes that were used to form part of the inclusion criteria for this study.

| **Appendix 1: AIS and ICD-10-AM diagnosis codes for thoracic and lumbar spinal cord injury** | |
| --- | --- |
| **AIS thoracic spine codes** | |
| 640400.3 | Cord contusion [includes the diagnosis of compression, or epidural or subdural haemorrhage within spinal canal documented by imaging studies or autopsy] |
| 640401.3 | Cord contusion with transient neurological signs (paraesthesia) but NFS as to fracture/dislocation |
| 640402.3 | Cord contusion with no fracture or dislocation |
| 640404.3 | Cord contusion with fracture |
| 640406.3 | Cord contusion with dislocation |
| 640408.3 | Cord contusion with both fracture and dislocation |
| 640410.4 | Incomplete cord syndrome (preservation of some sensation or motor function; includes anterior cord, central cord, lateral cord (Brown-Sequard) syndromes) but NFS as to fracture/dislocation |
| 640412.4 | Incomplete cord syndrome with no fracture or dislocation |
| 640414.4 | Incomplete cord syndrome with fracture |
| 640416.4 | Incomplete cord syndrome with dislocation |
| 640418.4 | Incomplete cord syndrome with both fracture and dislocation |
| 640420.5 | Complete cord syndrome (paraplegia with no sensation) but NFS as to fracture/dislocation |
| 640422.5 | Complete cord syndrome with no fracture or dislocation |
| 640424.5 | Complete cord syndrome with fracture |
| 640426.5 | Complete cord syndrome with dislocation |
| 640428.5 | Complete cord syndrome with both fracture and dislocation |
| 640442.5 | Cord laceration NFS [includes penetrating injury, transection or crush] |
| 640442.5 | Cord laceration incomplete (preservation of some sensation or motor function), but NFS as to fracture/dislocation |
| 640444.5 | Cord laceration with no fracture or dislocation |
| 640446.5 | Cord laceration with fracture |
| 640448.5 | Cord laceration with dislocation |
| 640450.5 | Cord laceration with both fracture and dislocation |
| 640460.5 | Complete cord syndrome NFS (paraplegia with no sensation or motor function) but NFS as to fracture/dislocation |
| 640462.5 | Complete cord syndrome with no fracture or dislocation |
| 640464.5 | Complete cord syndrome with fracture |
| 640466.5 | Complete cord syndrome with dislocation |
| 640468.5 | Complete cord syndrome with both fracture and dislocation |
| 650403.3 | Disc herniation with root damage (radiculopathy) |
| 630499.2 | Nerve root injury, single or multiple NFS |
| 630402.2 | Nerve root contusion; stretch injury |
| 630404.2 | Nerve root laceration NFS |
| 630406.2 | Nerve root laceration single nerve root |
| 630408.3 | Nerve root laceration multiple nerve roots |
| 630410.2 | Nerve root avulsion NFS |
| 630412.2 | Nerve root avulsion single nerve root |
| 630414.3 | Nerve root avulsion multiple nerve roots |
| **AIS lumbar spine codes** | |
| 630600.3 | Cauda equina contusion, NFS |
| 630602.3 | Cauda equina contusion with transient neurological signs but NFS as to fracture/dislocation |
| 630604.3 | Cauda equina contusion with transient neurological signs with no fracture/dislocation |
| 630606.3 | Cauda equina contusion with transient neurological signs with fracture |
| 630608.3 | Cauda equina contusion with transient neurological signs with dislocation |
| 630610.3 | Cauda equina contusion with transient neurological signs with fracture and dislocation |
| 631620.3 | Incomplete cauda equina syndrome but NFS as to fracture/dislocation |
| 630622.3 | Incomplete cauda equina syndrome with no fracture or dislocation |
| 630624.3 | Incomplete cauda equina syndrome with fracture |
| 630626.3 | Incomplete cauda equina syndrome with dislocation |
| 630628.3 | Incomplete cauda equina syndrome with fracture and dislocation |
| 630630.4 | Complete cauda equina syndrome but NFS as to fracture/dislocation |
| 630632.4 | Complete cauda equina syndrome with no fracture/dislocation |
| 630634.4 | Complete cauda equina syndrome with fracture |
| 630636.4 | Complete cauda equina syndrome with dislocation |
| 630638.4 | Complete cauda equina syndrome with both fracture and dislocation |
| 640600.3 | Cord contusion [includes the diagnosis of compression, or epidural or subdural haemorrhage within spinal canal documented by imaging studies or autopsy] |
| 640601.3 | Cord contusion with transient neurological signs (paraesthesia), but NFS as to fracture/dislocation |
| 640602.3 | Cord contusion with no fracture or dislocation |
| 640604.3 | Cord contusion with fracture |
| 640606.3 | Cord contusion with dislocation |
| 640608.3 | Cord contusion with both fracture and dislocation |
| 640610.4 | Incomplete cord syndrome (preservation of some sensation or motor function; includes lateral cord (brown-Sequard) syndrome) but NFS as to fracture/dislocation |
| 640612.4 | Incomplete cord syndrome with no fracture of dislocation |
| 640614.4 | Incomplete cord syndrome with fracture |
| 640616.4 | Incomplete cord syndrome with dislocation |
| 640618.4 | Incomplete cord syndrome with both fracture and dislocation |
| 640620.5 | Complete cord syndrome (paraplegia with no sensation) but NFS as to fracture/dislocation |
| 640622.5 | Complete cord syndrome with no fracture or dislocation |
| 640624.5 | Complete cord syndrome with fracture |
| 640626.5 | Complete cord syndrome with dislocation |
| 640628.5 | Complete cord syndrome with both fracture and dislocation |
| 640640.5 | Cord laceration NFS [includes penetrating injury, transection or crush] |
| 640642.5 | Cord laceration incomplete (preservation of some sensation or motor function), but NFS as to fracture/dislocation |
| 64064 | Cord laceration with no fracture or dislocation |
| 640646.5 | Cord laceration with fracture |
| 640648.5 | Cord laceration with dislocation |
| 640650.5 | Cord laceration with both fracture and dislocation |
| 640660.5 | Complete cord syndrome NFS (paraplegia with no sensation or motor function) but NFS as to fracture/dislocation |
| 640662.5 | Complete cord syndrome with no fracture or dislocation |
| 640664.5 | Complete cord syndrome with fracture |
| 640666.5 | Complete cord syndrome with dislocation |
| 640668.5 | Complete cord syndrome with both fracture and dislocation |
| 650603.3 | Disc herniation with root damage (radiculopathy) |
| 630699.2 | Nerve root or sacral plexus, single or multiple NFS |
| 630660.2 | Nerve root or sacral plexus contusion; stretch injury |
| 630662.2 | Nerve root or sacral plexus laceration NFS |
| 630664.2 | Nerve root or sacral plexus laceration single nerve root |
| 630666.3 | Nerve root or sacral plexus laceration multiple nerve roots |
| 630668.2 | Nerve root or sacral plexus avulsion NFS |
| 630612.2 | Nerve root or sacral plexus avulsion single nerve root |
| 630614.3 | Nerve root or sacral plexus avulsion multiple nerve roots |
| **ICD-10-AM diagnosis codes** | |
| S24.0 | Concussion and oedema of thoracic spinal cord |
| S24.10 | Injury of thoracic spinal cord unspecified |
| S24.11 | Complete lesion of thoracic spinal cord |
| S24.12 | Incomplete cord syndrome of thoracic spinal cord |
| S24.2 | Injury of nerve root of thoracic spine |
| S24.70 | Functional spinal cord injury, thoracic level unspecified |
| S24.73 | Functional spinal cord injury, T4/T5 |
| S24.74 | Functional spinal cord injury, T6/T7 |
| S24.75 | Functional spinal cord injury, T8/T9 |
| S24.76 | Functional spinal cord injury, T10/T11 |
| S24.77 | Functional spinal cord injury, T12 |
| S34.0 | Concussion and oedema of lumbar spinal cord [conus medullaris] |
| S34.1 | Other injury of lumbar spinal cord [conus medullaris] |
| S34.2 | Injury of nerve root of lumbar and sacral spine |
| S34.3 | Injury of cauda equina |
| S34.70 | Functional spinal cord injury, lumbar level unspecified |
| S34.71 | Functional spinal cord injury, L1 |
| S34.72 | Functional spinal cord injury, L2 |
| S34.73 | Functional spinal cord injury, L3 |
| S34.74 | Functional spinal cord injury, L4 |
| S34.75 | Functional spinal cord injury, L5 |
| S34.76 | Functional spinal cord injury, sacrum |

Appendix 2: Mixed effect regression modelling

| Mixed-effects logistic regression of LMN mobility on time and spinal surgery | | | |
| --- | --- | --- | --- |
| Number of groups | | 34 | |
| Number of observations | | 92 | |
| Variable | Odds ratio | P value | 95% CI |
| 6 months | 1 | (base) | (base) |
| 12 months | 0.72 | 0.62 | (0.1928219, 2.669069) |
| 24 months | 0.82 | 0.77 | (0.2206833, 3.076147) |
| Nospinal operation | 1 | (base) | (base) |
| Had spinal operation | 0.55 | 0.55 | (0.0750854, 3.969555) |
| PatientID (Vars_cons) | 2.95 |  | (0.6601761, 13.15547) |

| Mixed-effects logistic regression of LMN pain/discomfort on time and spinal surgery | | | |
| --- | --- | --- | --- |
| Number of groups | | 34 | |
| Number of observations | | 92 | |
| Variable | Odds ratio | P value | 95% CI |
| 6 months | 1 | (base) | (base) |
| 12 months | 1.19 | 0.85 | (0.2095966, 6.726843) |
| 24 months | 0.52 | 0.43 | (0.1030519, 2.64545) |
| No spinal operation | 1 | (base) | (base) |
| Had spinal operation | 0.75 | 0.82 | (0.0599512, 9.385371) |
| PatientID (Vars_cons) | 18.11 | **0.045** | (1.064189, 308.0873) |

| Mixed-effects logistic regression of LMN anxiety/depression on time and spinal surgery | | | |
| --- | --- | --- | --- |
| Number of groups | | 34 | |
| Number of observations | | 91 | |
| Variable | Odds ratio | P value | 95% CI |
| 6 months | 1 | (base) | (base) |
| 12 months | 0.49 | 0.33 | (0.1162847, 2.04941) |
| 24 months | 0.15 | **0.020** | (0.0308172, 0.7382962) |
| No spinal operation | 1 | (base) | (base) |
| Had spinal operation | 1.28 | 0.84 | (0.1154643, 14.17034) |
| PatientID (Vars_cons) | 5.88 |  | (1.549527, 22.30563) |

| Mixed-effects logistic regression of LMN selfcare on time and spinal surgery | | | |
| --- | --- | --- | --- |
| Number of groups | | 34 | |
| Number of observations | | 92 | |
| Variable | Odds ratio | P value | 95% CI |
| 6 months | 1 | (base) | (base) |
| 12 months | 1.17 | 0.83 | (0.2685838, 5.134737) |
| 24 months | 0.74 | 0.68 | (0.1703966, 3.173786) |
| No spinal operation | 1 | (base) | (base) |
| Had spinal operation | 0.99 | 0.99 | (0.0697516, 14.06569) |
| PatientID (Vars_cons) | 8.27 |  | (2.093711, 32.67381) |

| Mixed-effects logistic regression of LMN usual activities on time and spinal surgery | | | |
| --- | --- | --- | --- |
| Number of groups | | 34 | |
| Number of observations | | 92 | |
| Variable | Odds ratio | P value | 95% CI |
| 6 months | 1 | (base) | (base) |
| 12 months | 0.43 | 0.27 | (0.0934259, 1.955186) |
| 24 months | 0.88 | 0.88 | (0.1770309, 4.37645) |
| No spinal operation | 1 | (base) | (base) |
| Had spinal operation | 1.53 | 0.69 | (0.1948521, 11.98955) |
| PatientID (Vars_cons) | 3.10 |  | (0.5612175, 17.07438) |

| Mixed-effects logistic regression of UMN mobility on time and spinal surgery | | | |
| --- | --- | --- | --- |
| Number of groups | | 883 | |
| Number of observations | | 2,222 | |
| Variable | Odds ratio | P value | 95% CI |
| 6 months | 1 | (base) | (base) |
| 12 months | 0.57 | **0.002** | (0.4019375, 0.8215574) |
| 24 months | 0.53 | **0.001** | (0.3632245, 0.7664044) |
| No spinal operation | 1 | (base) | (base) |
| Had spinal operation | 1.19 | 0.55 | (0.679857, 20.077769) |
| PatientID (Vars_cons) | 23.36 |  | (15.0571, 36.24875) |

| Mixed-effects logistic regression of UMN pain/discomfort on time and spinal surgery | | | |
| --- | --- | --- | --- |
| Number of groups | | 882 | |
| Number of observations | | 2,213 | |
| Variable | Odds ratio | P value | 95% CI |
| 6 months | 1 | (base) | (base) |
| 12 months | 0.85 | 0.36 | (0.6033373, 1.199372) |
| 24 months | 0.69 | **0.046** | (0.4853988, 0.9936765) |
| No spinal operation | 1 | (base) | (base) |
| Had spinal operation | 2.34 | **0.001** | (1.426333, 3.828459) |
| PatientID (Vars_cons) | 8.65 |  | (6.096883, 12.2601) |

| Mixed-effects logistic regression of UMN anxiety/depression on time and spinal surgery | | | |
| --- | --- | --- | --- |
| Number of groups | | 881 | |
| Number of observations | | 2,209 | |
| Variable | Odds ratio | P value | 95% CI |
| 6 months | 1 | (base) | (base) |
| 12 months | 1.00 | 0.98 | (0.7596675, 1.325574) |
| 24 months | 0.98 | 0.87 | (0.7300853, 1.306587) |
| No spinal operation | 1 | (base) | (base) |
| Had spinal operation | 1.21 | 0.35 | (0.8090583, 1.824283) |
| PatientID (Vars_cons) | 5.83 |  | (4.439328, 7.651102) |

| Mixed-effects logistic regression of UMN selfcare on time and spinal surgery | | | |
| --- | --- | --- | --- |
| Number of groups | | 883 | |
| Number of observations | | 2,226 | |
| Variable | Odds ratio | P value | 95% CI |
| 6 months | 1 | (base) | (base) |
| 12 months | 0.73 | **0.037** | (0.5409274, 0.9812715) |
| 24 months | 0.58 | **0.001** | (0.422522, 0.791895) |
| No spinal operation | 1 | (base) | (base) |
| Had spinal operation | 0.98 | 0.93 | (0.6150952, 1.560716) |
| PatientID (Vars_cons) | 8.74 |  | (6.582077, 11.59765) |

| Mixed-effects logistic regression of UMN usual activities on time and spinal surgery | | | |
| --- | --- | --- | --- |
| Number of groups | | 882 | |
| Number of observations | | 2,224 | |
| Variable | Odds ratio | P value | 95% CI |
| 6 months | 1 | (base) | (base) |
| 12 months | 0.65 | **0.009** | (0.4742324, 0.8972157) |
| 24 months | 0.39 | **0.000** | (0.2822198, 0.5485699) |
| No spinal operation | 1 | (base) | (base) |
| Had spinal operation | 1.28 | 0.24 | (0.8449396, 1.946816) |
| PatientID (Vars_cons) | 5.37 |  | (3.904297, 7.373171) |
